# Highly transparent light emitting diodes on graphene encapsulated Cu nanowires network

**DOI:** 10.1038/s41598-018-31903-7

**Published:** 2018-09-13

**Authors:** Youyang Huang, Zongxing Huang, Zhibai Zhong, Xu Yang, Qiming Hong, Huachun Wang, Shengrong Huang, Na Gao, Xiaohong Chen, Duanjun Cai, Junyong Kang

**Affiliations:** 1 Fujian Key Laboratory of Semiconductor Materials and Applications, CI center for OSED, College of Physical Science and Technology, Xiamen University, Xiamen, 361005 China; 2 College of Chemistry and Chemical Engineering, Xiamen University, Xiamen, 361005 China; 3 Xiamen Top-succeed Electronics Technology Co. Ltd, Xiamen, 361022 China; 4 Department of Chemistry, Duke University, Durham, NC 27708-0354 USA

## Abstract

The internal quantum efficiency of blue LEDs is almost close to the limit, therefore, advanced transparent electrode has been long explored for gaining high external quantum efficiency. However, work function mismatch at electrode-semiconductor interface remains the fundamental difficulty in obtaining low resistance ohmic contact. Here, we demonstrate the gas phase encapsulation of graphene layer on superfine Cu nanowires network by chemical vapor deposition for highly transparent LEDs. The fast encapsulation of graphene shell layer on Cu nanowires achieves high optoelectronic performance (33 Ω/sq @ 95% T), broad transparency range (200~3000 nm) and strong antioxidant stability. A novel phenomenon of scattered-point contact is revealed at the Cu nanowires/GaN interface. Point discharge effect is found to produce locally high injection current through contact points, which can effectively overcome Schottky barrier and form ohmic contact. The transparent LED on Cu@graphene nanowire network is successfully lighted with bright blue emission.

## Introduction

**Figure 1:**
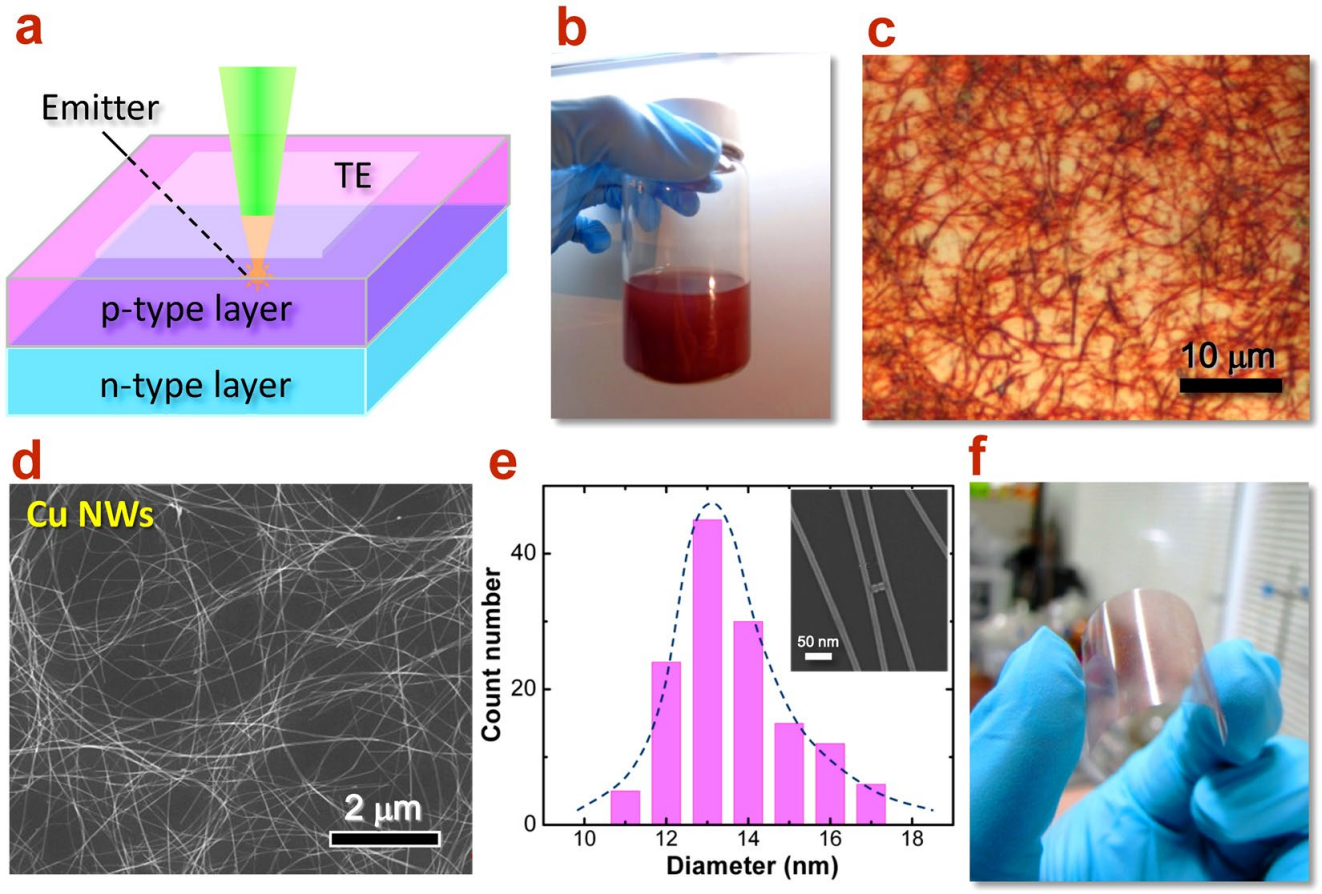
Superfine Cu NWs as TE materials with high-aspect-ratio. (**a**) Schematic of light emitting device integrated with TE for light transmission, (**b**) photo of superfine Cu NWs ink, and (**c**) optical microscope image and (**d**) SEM image of Cu NWs. (**e**) Diameter distribution of Cu NWs. The inset is the SEM image of well dispersed Cu NWs, which shows the uniformity and superfine diameter. (**f**) Cu NWs network imprinted on PET film, which possesses high transparency and flexibility.

Group III-nitrides have been promising semiconductor materials for a wide range of technological applications in optoelectronics such as high power, high frequency, and high temperature electronic devices^1–4^. The success in the fabrication of InGaN based blue light emitting diodes (LEDs) pushed the rapid progress of optoelectronic devices^5^. As we know, the electro-optical conversion efficiency of a LED could be evaluated by the external quantum efficiency (η_e_), which is further dominated by two factors, internal quantum efficiency and light extraction efficiency^6^. After decade’s effort, the internal quantum efficiency of blue light LED has been largely improved and almost close to the limit (>80%)^7^. Thus, the improvement of light extraction efficiency was long highly concerned in optoelectronic devices and now becomes extremely crucial. In order to obtain better light extraction efficiency, transparent electrode (TE), as an important role in the device structure (Fig. 1a), has been widely studied in the field of materials and devices^8–10^. A perfect combination of TEs with LED strongly requires multiple superior properties such as high transparency, low resistance, low cost, high flexibility, high reliability, ohmic contact and etc., which apparently is a big challenge.

So far, the most promising TE materials include transparent conductive oxides, carbon nanotubes (CNTs)^11^, metal nanowire (NW)^12^ networks, and graphene^13^. Indium tin oxide (ITO) is regarded as the best transparent conductive oxide layer with the lowest resistivity on a commercial scale^14^. However, the high price owing to the lack of indium resources calls the replacement of ITO by novel advanced materials. The percolated metal NW networks seem to well meet most of the requirements of an excellent TE, including good electrical and optical performances, industrial mass synthesis and low cost (the price of Cu is only 1/100 of ITO or Ag)^15^. Cai and his coworkers recently achieved the super-fine Cu NWs with high optoelectronic performance of 51 Ohm/sq at 93% transmittance, almost comparable to ITO^16^. However, for the application of Cu NWs in LEDs, the stability against oxidation and the low resistance contact are two most harsh bottlenecks and conflicts in the way forward. Recently, methods such as nickel or silver coating have been proposed to prevent Cu NWs from oxidation^17,18^. While, it was found that the sheet resistance would increase with increasing the nickel percentage^17,19,20^ and the contact between Cu NWs and active materials, e.g., GaN layer, would be largely affected by the coated shell layer. Hence, new technique is strongly desired to balance or even eliminate these inevitable conflicts.

In this work, we propose a chemical vapor deposition (CVD) method for fast encapsulation of graphene shell layer on Cu NWs network TEs for achieving ohmic contact on GaN based LED chips. Ultra-long (>40 μm) and super-fine (~13 nm) Cu NWs were synthesized via a solution method. The gas phase encapsulation of graphene was used to effectively improve the optoelectronic performances of the Cu NWs network as well as resist oxidation. By using pre-annealing treatment followed by graphene encapsulation, the Cu@graphene NW TEs could be fabricated into GaN-based LED chips on wafer and ohmic contact was perfectly achieved. As a result, a completely transparent LED was successfully lighted giving bright blue emission. Simulation and electroluminescence (EL) investigations were carried out for demonstrating a unusual contact mode in NW network electrode.

## Results and Discussion

### High-aspect-ratio Cu NWs as transparent conductor

Superfine Cu NWs in high aspect ratio were synthesized through a solution-processed method with nickel catalyst (See Methods section)^21^. The as-synthesized Cu NWs were stored in organic solution (such as hexane) isolated from air, as an ink, to keep them from oxidation and maintain the dispersibility. In this way, this Cu NWs ink can be kept in the air without any deterioration for more than two months, remaining in original reddish color (Fig. 1b). To characterize the structural properties and uniformity, the Cu NWs were transferred onto a Si substrate via vacuum filter method. Figure 1c shows the optical micrograph of the Cu NWs, indicating the excellent dispersive property. The average length of Cu NWs has been determined to be 40~50 μm (Fig. S1). From Fig. 1d, the scanning electron microscope (SEM) image shows the microscopic morphology of the Cu NWs. One can see that the Cu NWs with uniform diameter are well distributed as a network and with very few nanoparticles, which do not have significant influence on the transparency or electrical property of TEs. By measuring more than 200 NWs, the diameter distribution is plot in Fig. 1e, which reveals an average diameter of 13.5 ± 2 nm. Based on these geometric data, the aspect ratio can be obtained in the level of larger than 3000, which is the finest Cu NW ever reported. In principles, the higher the aspect ratio of Cu NWs is, the better its optoelectronic properties should be. Thanks to the high aspect ratio of these Cu NWs, the performance of the fabricated transparent conductive film reaches 50 Ω/sq at 93% transmittance and has an excellent transparency from 200 to 3000 nm (Fig. S2).

The Cu NWs ink can be easily imprinted as electrodes in various shapes, patterns, and sizes with designed templates. For the application on semiconductor devices, a 2″ size wafer has been uniformly covered with complete Cu NWs conducting film. Moreover, the ink could be imprinted onto various substrates, including flexible substrates such as PET or polyimide (PI) paper, as shown in Fig. 1f. The excellent transparency and flexibility of this Cu NWs network promises the potential for broad applications in advanced optoelectronic devices. However, the narrowest bottleneck the Cu NWs meet is the poor anti-oxidant ability when working in the air (Figs S3–4).

### Gas phase encapsulation of graphene shell on Cu NW network

**Figure 2:**
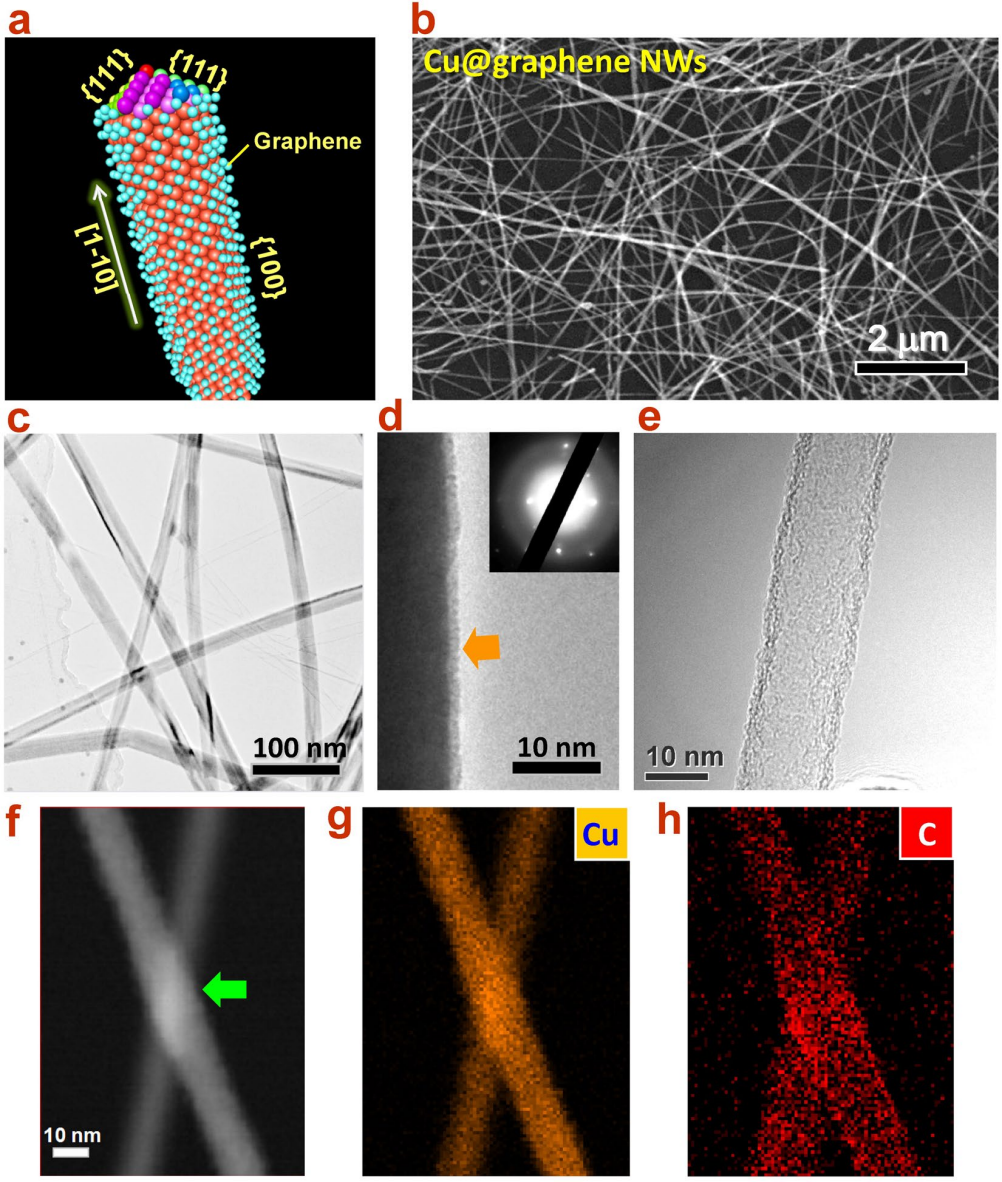
Graphene encapsulation on Cu NWs. (**a**) Atomic structure of Cu@graphene NW, where the graphene shell layer is encapsulated on the {100} sidewall of Cu NW. (**b**) SEM image, (**c**) TEM image, and (**d**) high resolution TEM image of Cu@graphene NW. The inset is the SAED image showing the growth direction and structure of Cu@graphene NW. (**e**) TEM image of free-standing graphene nanotube after the removal of Cu core. (**f**) Scanning TEM image of interlaced Cu@graphene NWs. The junction is firmly fused after nano-welding treatment. (**g**) and (**h**) Elemental mapping images of Cu and C, respectively.

In order to improve the ability of oxidation resistance without additional unwanted deterioration in conductivity, we propose a gas phase method for the encapsulation of graphene shell directly on Cu NWs network (Fig. 2a). Since graphene is an atomically thin and conductive layer, the encapsulation is believed to protect Cu NWs against the oxygen attack meanwhile preserve the high transmittance and conductivity. According to this idea, the encapsulation of graphene was carried out in a process with low pressure CVD system (See Methods and Supporting information, Fig. S5). Firstly, the Cu NWs network was imprinted onto target substrate, e.g., GaN wafer, and then put in a Cu foil pocket (Fig. S6), which is designed for the protection of Cu NWs against melting under high temperature^21^. Secondly, the CVD heating zone was heated up to reaction temperature, 700 °C and the precursor gases (CH_4_ and H_2_) were injected into the chamber. Thirdly, the sample was pushed into the heating zone using magnetic slider for 1~5 min and then, pulled away for rapid cooling. Finally, the graphene shell layer could be successfully encapsulated onto the sidewall surface of Cu NWs network (Fig. 2b). As we know, Cu has a face-centered cubic (fcc) crystalline structure. The sidewall surface of the Cu NW is constructed by {100} facets and the top surfaces are exposed with {111} facets^16^. Hence, a Cu NW usually grows along the [1$\overline{{\rm{1}}}$0] direction and forms a five-fold twinned pentagonal structure, which could be determined from SAED pattern (inset of Fig. 2d). The graphene layer then is easily deposited on the {100} sidewall surface (Fig. 2a) and forms complete capsule through gas phase method. From Fig. 2b, the SEM image of the Cu@graphene NWs network shows the uniform, dispersive, smooth, and intact morphology.

In order to further confirm the graphene encapsulation, transmission electron microscope (TEM), energy-dispersive X-ray spectroscopy (EDS), X-ray photoelecton spectroscopy (XPS) and Raman investigations were conducted systematically. From the TEM image in Fig. 2c, one can see that the Cu@graphene NWs network remains ultrathin and uniform diameter with smooth surface. Detailed examination (Fig. 2d) reveals that the sidewall surface is encapsulated with a ultrathin layer of graphene shell in uniform thickness of about 1.8 nm, which corresponds to 5 ~ 6 graphene layers (Figs S7–8)^22^. Element mappings of Cu and C on two interlaced Cu@graphene NWs were carried out, as shown in Fig. 2f,g. It can be observed from Figs 2f and S9 that the connecting junction of Cu NWs has been fused after high temperature annealing, which is important for the accomplishment of electrical conduction throughout entire network. It can be seen that Cu appears in the core area of the NWs (Fig. 2d,g), whereas the shell layer is covered by C (graphene layer). This strongly confirms the formation of graphene shell layer and the full coverage on entire sidewall surface. As a test, we attempted to anneal the Cu@graphene NW at even high temperature (900 °C) under vacuum for 15 min. After this treatment, it is interestingly found in Fig. 2e that due to the evaporation of Cu atoms, the Cu core of NW has been removed and the graphene nanotube left. This nanotube actually is the graphene capsule, which could exist independently without the support by the Cu core. This result further reveals the morphology and mechanical stability of the graphene shell and meanwhile indicates another novel method for producing micron-long and nanometer-thick graphene nanotubes network. In addition, the XPS measurement of C_1s_ spectrum (Fig. S10) demonstrated a *sp*^2^/*sp*^3^ ratio of about 3.41 (*sp*^2^ = 77%), confirming the domination of the graphene C-C bonding. Raman spectra (Fig. S11) were surveyed on areas of Cu@graphene NWs and bare Si substrate, respectively, for comparison. The results showed that a sharp G band at about 1580 cm^−1^ and clear D peak (~1270 cm^−1^), indicating the sp^2^ C-C bonds^23^. Whereas, On the bare Si surface only characteristic peaks of Si at 521 cm^−1^ and 964 cm^−1^ (second order peak) can be observed, which means that the growth of graphene only takes place on the sidewall surface of Cu NWs other than the entire coverage on the whole substrate.

### High performances and robust stability of Cu@graphene NW network

**Figure 3:**
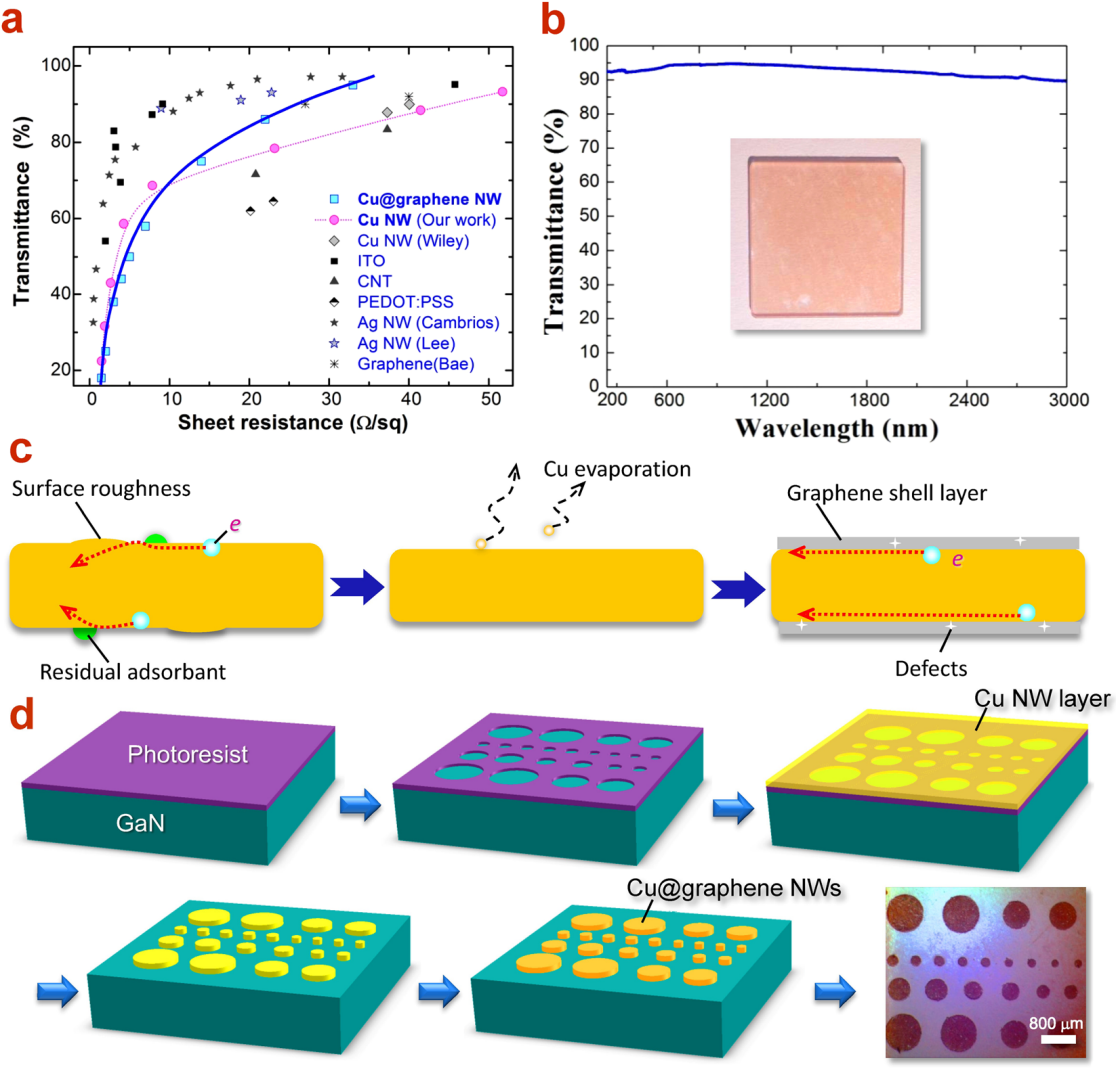
Optoelectronic performances of Cu@graphene NWs TEs. (**a**) Transmittance as a function of sheet resistance for various advanced TE materials^13,36–41^. (**b**) Optical transmittance spectrum of Cu@graphene NWs film. The inset is the photograph of the Cu@graphene TE film on quartz glass slice. (**c**) Schematics of electron transport on surface and interface along the Cu@graphene NW. (**d**) Processing steps of photolithographic patterning of Cu@graphene NW TEs on semiconductor wafer. The last image is the optical micrograph of patterned Cu@graphene NW TE pads in various sizes.

Owing to the excellent optoelectronic properties of graphene, the Cu@graphene NWs TEs achieve simultaneously the low sheet resistance, which is even better than the original Cu NWs^24^, as summarized in Fig. 3a. The Cu@graphene NWs network was imprinted on quartz glass and the UV-Visible-infrared transmission spectrum was recorded, as shown in Fig. 3b. The TE possesses a relatively high transmittance (>90% @ 25 Ω/sq) over the entire wavelength range from deep ultraviolet to near infrared (200~3000 nm). Such a wide range of transmittance indicates that the Cu@graphene NWs could be widely applied for optoelectronic devices in various wavelengths. In particular, the transmittance of the Cu@graphene NWs network after graphene encapsulation increased by about 17% compared to the pure Cu NWs network. Usually the two-dimensional graphene film has a certain light absorption in the visible ultraviolet band (<400 nm), but because of its 3D-shaped encapsulation on the surface of the Cu NWs network, the actual effective area of light absorption becomes negligibly small. On the other hand, as mentioned previously, the Cu NWs will easily get melted under high temperature (>700 °C) in vacuum which is lower than the melting point of bulk Cu (1090 °C). This is attributed to the influence by the nano-structured conformation of Cu NWs^21^. Hence, in the process of graphene coating, although the Cu foil pocket was used to protect Cu NWs from melting, the surface atoms of Cu NWs could still be volatilized at high temperature, which makes the diameter slightly smaller (Fig. S12). Thus, the transmittance of Cu@graphene NW network is effectively improved after graphene encapsulation, and meanwhile the transmittance curve becomes more flat.

In principle, the charge transport through a metal NW and its network mainly goes along the surface of NWs, as indicated in Fig. 3c. The transport of electrons, for example, on the surface of Cu@graphne NWs is likely to follow these steps: (1) The pure Cu NWs network achieves its conductivity only after annealing treatment. Cu NWs could then become smooth and conductive, which is mainly due to the effective nano-welding of connecting junctions (Fig. S9) and the removal of the surface adsorbents. However, there may still remain some residual adsorbents, affecting the Cu NWs sidewall surface smoothness. This type of non-smooth surface will cause the electron scattering to some degree and consequently, lead to the limitation of conductivity; (2) During the graphene encapsulation process at high temperature (700 °C), the evaporation of Cu atoms from the outmost sidewall surface of Cu NWs will reduce the diameter of the NWs as well as the surface roughness (see supporting information, Fig. S14). Meanwhile, the complete removal of oleylamine residues was also achieved during the graphene encupasulation. As a result, this smoothening will effectively minimize the electron scattering by the surface roughness and enhance the conductivity of Cu NWs network. (3) After the coating of the graphene shell layer, the sidewall surface of Cu NWs is further modified. The electron will move along the Cu-graphene interface rather than in the graphene shell. Thus, the protection and modification by graphene layer could effectively enhance the mobility of electrons on the surface of Cu NWs, thereby reducing the overall resistance. As a result, the graphene-encapsulated TCE has a lower sheet resistance for an equivalent transparency, as shown in Fig. 3a. On the other hand, since the defects and grain boundaries within the graphene shell layer are not highly involved in the electron transport^25^, the electrical properties of the Cu@graphene NWs network will not be deteriorated.

Ultimately, Cu@graphene NW TEs achieves an optoelectronic performance of 33 Ω/sq @ 95% transmittance, which has been comparable to ITO and Ag NWs, as shown in Fig. 3a. We found that along this T ~ R_s_ curve, the optoelectronic behavior of Cu NWs and Cu@graphene NWs has a crucial turning point at around 8 Ω/sq. To the lower resistance side, the transmittance of TEs decreases rapidly whereas to the higher resistance side, the transparency increases slowly and gradually saturates. The similar character and same turning point has been observed in other TE materials such as ITO and Ag NW. It could be described by the Grüner-Coleman metric^13,26^:

1 $${\rm{T}}={(1+\frac{188.5}{{R}_{s}}\frac{{\sigma }_{op}}{{\sigma }_{dc}})}^{-2}$$

where T stands for the transmittance, R_s_ refers to the sheet resistance, σ_dc_ is the dc conductivity, and σ_op_ is the optical conductivity. By fitting the T ~ R_s_ curves with eq. (1), the σ_dc_/σ_op_ ratio could be estimated, which presents the figure of merit (FOM) of TEs. In principles, the higher FOM value indicates the better TE performance. The FOM of the pure Cu NWs is determined to be 111 and that of Cu@graphene NWs gets 121. The improvement of performance after graphene encapsulation appears mainly on the right hand side to the turning point, which could be attributed to the modification of Cu surface and the formation of Cu-graphene interface.

The Cu@graphene NWs exhibit distinguishing antioxidant ability even under high temperature or extreme conditions. The Cu@graphene NWs network film was put in oven with high temperature (85 °C) and high humidity (85%) for 24 h (Fig. S13). The results showed excellent anti-oxidant ability with stable resistance (only slight increase of resistance (ΔR/R_0_ = 5%) after 5-day heating) and transmittance whereas the bare Cu NW network has been seriously oxidized. Another stability test was carried out by heating at 200 °C in air for 24 h (Fig. S14). The Cu@graphene NWs could well stand this extreme condition within 4 h without any resistance change. Long-term stability was examined as well under ambient for 2 months (Fig. S15) and the optoelectronic properties were well kept without deterioration. Such robust oxidation resistance is mainly attributed to the protection by the graphene shell layer, suggesting the strong capability for applications in optoelectronic devices such as LED or solar cell. In addition, for those common polymer substrates such as PET or PI films which could not stand high temperature treatment, a PDMS-assisted transfer of Cu@graphene NWs network could be used to make a flexible conductive film (see details in supporting information, Figs S16–S17).

### Ohmic contact between Cu@graphene NW network and GaN-based LED

Photolithography is an important technique for the fabrication of semiconductor devices^27^. Through the combination of specific mask templates, desired and complicated device structures could be easily fabricated into chips array on wafer, especially for the electrodes. In order to make the Cu@graphene NW TEs on LED chips, we combine the techniques of photolithography together with filter imprinting, as shown in Fig. 3d. Firstly, the test with a template including masks in various sizes (circle diameters from 10 μm to 800 μm) was conducted, Through the photolithography processes, a patterned Cu@graphene NW TE could be fabricated on GaN wafer. Most importantly, the nano-welding, the formation of ohmic contact, or even other special treatment on Cu NWs network could be carried out separately before the graphene shell coating.

From the micrograph in Fig. 3d, it can be confirmed that the patterning of Cu@graphene NW TEs in size from 10 μm up to 800 μm could be achieved. Such a wide range of TE size basically meets the production demand of various device chips, especially for the LED chips on wafer. On the other hand, the electrical contact between the Cu@graphene NW TEs and the GaN conducting layers was investigated. For the purpose of applications in optoelectronic devices, how to achieve low resistance contact to active semiconductor layers such as n-type and p-type conducting layers is the most critical issue.

In principles, it is rather difficult to achieve low resistance ohmic contact to both n-type and p-type GaN conducting layers with the same metal electrode. This interesting phenomenon implies a novel contact model and special contacting mechanism, which is believed to be related with nano-structured geometry of Cu NWs network. Firstly, the match of work functions between metal electrode and semiconductor is basic requirement for ohmic contact. Table 1 lists the work functions of commonly used metals and GaN semiconductor. The work function values of Cu are close to other electrode metals such as Ti, Au, Ni and Al. Therefore, the ohmic contact of Cu NWs to n-GaN is reasonable, attributed to the work function matching. However, the difference between the work function of Cu and the p-GaN appears rather large and should easily form Schottky barrier. Consequently, it should lead to a Schottky contact rather than an ohmic one. Why can the Cu@graphene NW TEs achieve ohmic contact to p-GaN with just a low temperature annealing? The possible explanation could be found from the morphology and structure of Cu NWs. As we know, the conventional metal electrode pad on optoelectronic devices is a continuous film, which forms a two-dimensional metal-semiconductor contact at the interface. When applying voltage, the current injection forms a uniform current flow across the interface. In contrast, though the Cu@graphene NWs constructs a two-dimensional network, from the microscopic point of view, the contact between the Cu NWs and the GaN surface should be scattered points other than a continuous plane. The structure and feature of this point contact would consequently lead to rather different property compared to that of conventional film electrodes.

**Table 1:** Work functions of metal materials and GaN semiconductor^31–35^.

Materials	Ti	Au	Ni	Pt	V	Al
Work function (eV)	4.33	5.1	5.04	5.12	4.3	4.28
**Materials**	**Cu (Cal.)**	**Cu (Exp.)**	**n-GaN**	**p-GaN**		
Work function (eV)	(100)~4.84 (110)~4.72 (111)~5.19	(100)~4.59 (110)~4.48 (111)~4.94	4.2	7.5		

**Figure 4:**
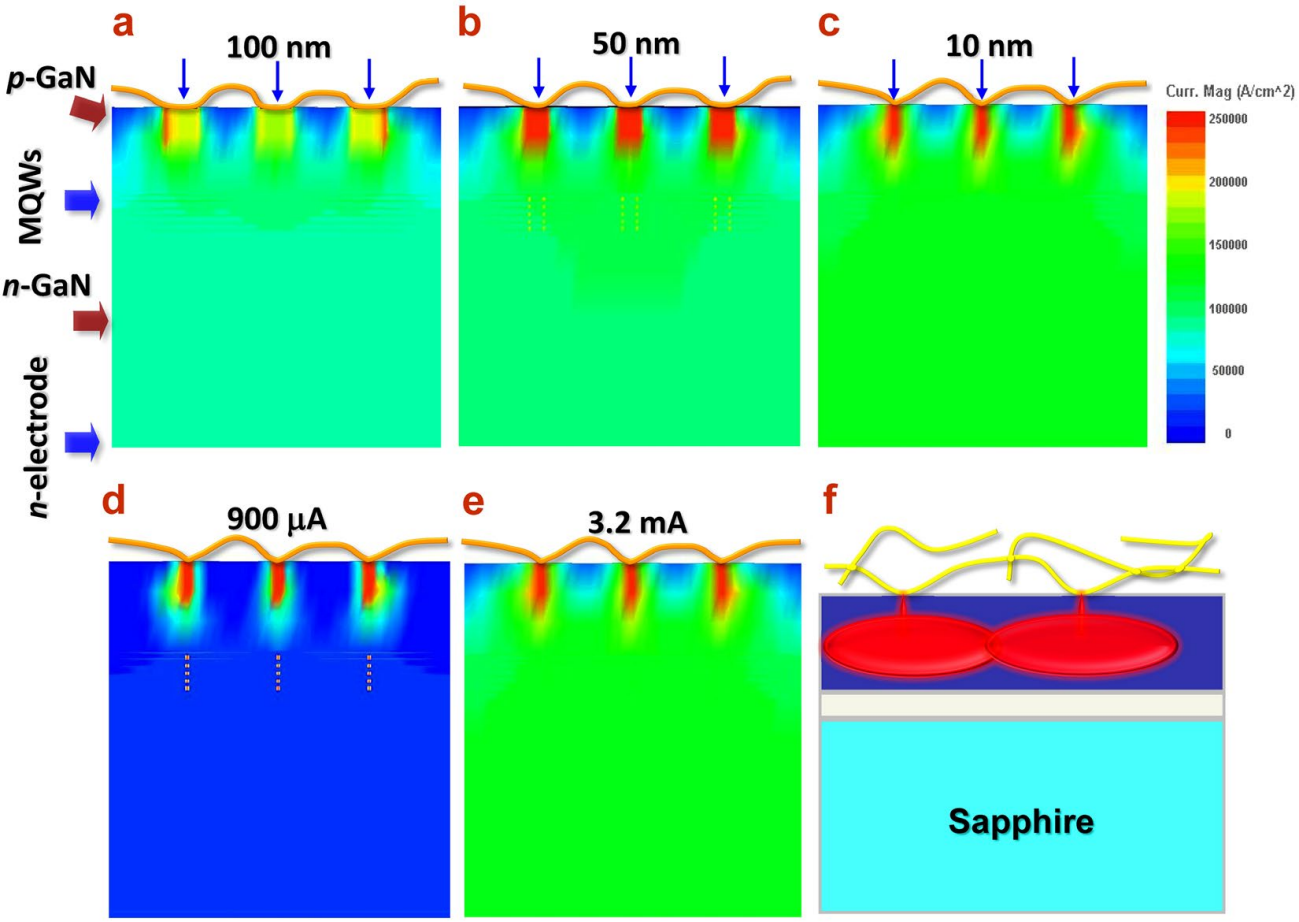
Current injection and distribution in LED structure with Cu NWs electrodes. Cross-sectional view of color contours of current distribution for Cu NWs in various contact point sizes of (**a**) 100 nm, (**b**) 50 nm and (**c**) 10 nm. Cu NWs are fabricated as the p-GaN electrode with scattered point contacts. Color contours of current distribution for the case of 10-nm contact point size under an injecting current of (**d**) 900 μA and (**e**) 3.2 mA. (**f**) Schematic of current injection and lateral spreading through the nanoscopic contact point at the Cu NW/GaN interface. The high density current injection through the contact point shows a point discharge behavior.

In order to make clear the current injection mechanism, we conducted a systematic investigation by using APSYS simulation package^28^. Models of GaN-based LED with Cu NW electrode were constructed by setting point contacts between Cu NWs and p-GaN layer. Various contacting sizes (diameter from 100 nm to 10 nm) with 100 nm separation were set and considered. By taking the difference of work function into account, a Schottky barrier of 3.05 eV was presumed between the Cu NW and the p-GaN layer and the current distribution was calculated, as shown in Fig. 4. It is interesting to see that as the size of the contact area decreases (Fig. 4a–c), the local density of the injection current underneath the Cu NW point becomes denser and meanwhile, the current from neighboring points well coalesces to form a uniform flow through the active layer of multi-quantum wells. This could be attributed to the point discharge effect at the narrow and sharp contact point, where the high current density results in a strong injection overcoming the barrier potential. Moreover, because the overall area of discrete contact points is much less than the entire electrode area, the total contact resistance along the current path could be much lower than the conventional film electrode. Now, one can finally understand why the low resistance ohmic contact could be achieved for this NWs network system even without work function matching. One the other hand, the influence by the current intensity (from 900 μA up to 3.2 mA) was investigated for the case of 10-nm contact point, as shown in Fig. 4d,e. One can see that at low current injection, the flow is only strongly trapped under the contact point and poor in lateral spreading. While, when the current increases up to 3.2 mA, the lateral flow well combined from neighboring points and consequently, forms the uniform and complete current distribution similar to the case of the conventional film electrode.

**Figure 5:**
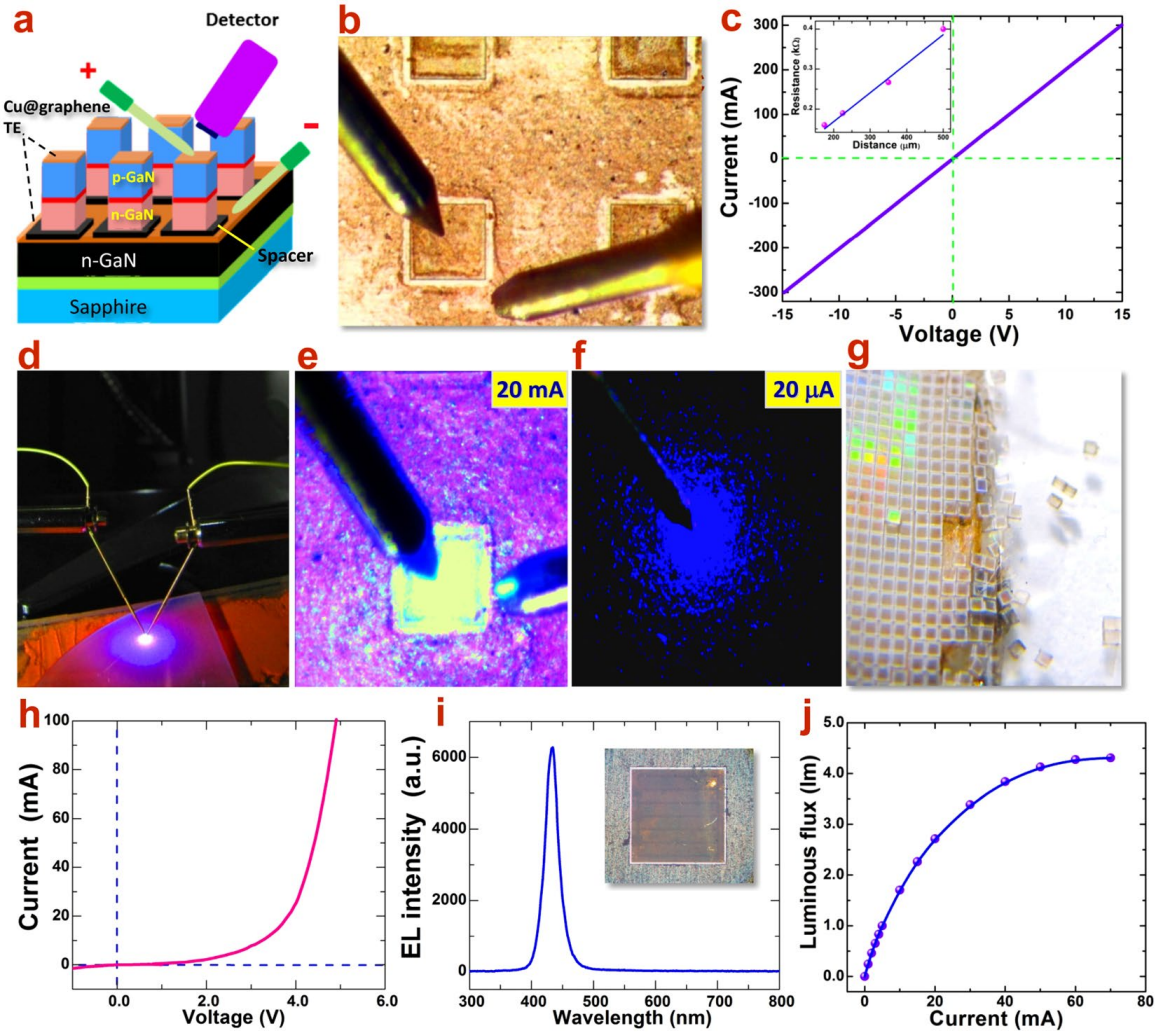
Transparent LED chips with Cu@graphene NWs electrodes. (**a**) Structure of GaN-based LEDs on Cu@graphene NW TEs. The placement of probes and detector is setup for electrical and optical measurements. (**b**) Optical micrograph of patterned LED chips with Cu@graphene NWs. The size of an individual chip is 300 μm by 300 μm. (**c**) I-V curve of the Cu@graphene NWs electrodes on p-GaN conducting layer. An ohmic contact behavior (linear) could be seen. The inset is the contact resistance as a function of electrode bar distance in the TLM model. (**d**) Photograph of EL emission of LED chip on Cu@graphene NWs TEs; and (**e**,**f**) optical micrographs of the EL emission of the transparent LED chip under working current of 20 mA and 20 μA, respectively. High transparency is achieved without any light blocking. (**g**) Photograph of laser-cut LED chips array from a 2″ wafer. (**h**,**i**) I-V curve and EL spectrum of the transparent LED. A pure and sharp emission at 437 nm is obtained. The inset shows a transparent LED chip in vertical structure by using Cu@graphene NWs TE. (**j**) Luminous flux as a function of current for the transparent LED.

Following the aforementioned fabrication steps, a series of rectangular-shaped Cu@graphene NW electrodes were made on both n- and p-GaN wafers and the transmission-line measurement (TLM) was conducted to determine the contact resistance between the electrode and GaN layers (see also Supporting Information, Fig. S18). After optimized annealing and graphene encapsulation, it is found that the I-V curves of Cu@graphene NW TEs on both n-type and p-type GaN layer show clear linear behavior, as shown in Figs 5c and S19, which strongly indicate the ohmic type contact to GaN layers. By the means of TLM method, resistances between pairs of rectangular TE bars in various distances were measured respectively and the plot of resistance versus contact separation can be obtained, as shown in the inset of Fig. 5c. By linear fitting, the contact resistance to n-GaN is determined to be 6 × 10^−4^ Ω cm^2^ and that to p-GaN is 4 × 10^−3^ Ω cm^2^, which is good enough and comparable to conventional ohmic electrodes^29^.

The above results reveal a novel electrode contact model for one-dimensional nanostructures. The locally high intensity of injecting current flow by the effect of the point discharge makes it possible to effectively overcome the Schottky barrier and leads to the effective vertical current injection as well as the lateral coalescence. This contact model could be especially useful for those metal-semiconductor systems in the absence of work function matching.

### Highly transparent LED on Cu@graphene NWs network

Since the ohmic contact to both n-GaN and p-GaN has been achieved, now it is ready to fabricate entire LED chips and characterize their performances. By combining photolithography and filter imprint method, the array of GaN based blue LED chips on board was fabricated with Cu@graphene NWs network as TEs, as shown in Fig. 5a,b. By this processing method, the Cu@graphene NW TEs was imprinted on LED chips, showing good uniformity and clear edges. Most importantly, the ohmic contact of Cu NW core could be achieved on the GaN surface before graphene encapsulation. And after graphene encapsulation, the Cu NWs network is further modified and protected. By laser dicing from the back side, individual LED chips was obtained and the TEs remain complete and clean (Fig. 5g). To test the property and performance, the as-prepared Cu@graphene-based LED chips were placed on the probe station for electrical and EL measurements (Fig. 5b). The positive and negative probes were approaching the p and n-type Cu@graphene NW TEs, respectively, and external voltage was applied for testing the LED working performances. As a result, the LED chip could be successfully lighted giving bright blue emission, as shown in Fig. 5d. The I-V curve was recorded as shown in Fig. 5h. The turn-on voltage is determined to be about 1.6 V, consistent with that with traditional electrode (Ti/Al, Ni/Au, about 2 V)^30^. It was confirmed that after 100 working cycles, the LED performance is stable and the I-V behavior remains unchanged (Fig. S20). For comparison, another control LED with ITO transparent current spreading layer ontop p-GaN was fabricated, which showed the turn-on voltage of 2.64 V (Figs S21–22). The ohmic TE with the Cu@graphene NWs network plays the key role in achieving the low contact resistance and good working voltage. The close-up EL photograph under 20 mA (Fig. 5e) shows that the blue light emission comes out from the entire LED chip surface without light blocking. This indicates the excellent current spreading over the whole p-type area and the excellent transparency of the Cu@graphene NWs network.

In order to further investigate the relationship between the current intensity and its lateral spreading, as aforementioned in the APSYS simulation, we gradually reduce the injection current and keep observing the light emission. As shown in Fig. 5f, it is found that when the current decreases down to 20 μA, diming blue light still could be emitted while the intensity becomes weaker. What is most interesting is that in the area away from the probe tip, the light emission turns non-continuous and forms scattered blue dots. This phenomenon provides important evidence supporting the model of point contacts and the effect of point discharge, as described previously. Since the contact of the Cu@graphene NWs with GaN forms scattered point-like pattern, the current injection firstly should go through the contact points. The bright blue dots correspond to those sites beneath the contact points where the QWs are activated and light emits. Under low current intensity, the tip injection of current is still strong while the lateral flow between adjacent contact points becomes rather weak. This finally results in poor current coalescence and discrete light emission. This phenomenon provides the clear evidence well consistent with the APSYS simulation results (Fig. 4e). As the current increases up to the typical value (20 mA), uniform lateral spreading can be re-obtained and continuous emission over entire chip area is achieved (Fig. 5e).

The optical properties of this LED chip were investigated by setting the spectrometer fiber over the Cu@graphene NWs TEs and the EL spectrum was recorded. From Fig. 5i, one can see that the central emission peak is located at about 437 nm in the blue region. The peak is sharp with a full width at half maximum (FWHM) of only 23 nm. No additional impurity related peak is observed, indicating the high stability and excellent light transmission with the Cu@graphene NW TEs. Figure 5j shows the luminous flux of the LED chip, which increases with the increasing of the inject current and saturates to 4.27 lm after 60 mA. Meanwhile, the luminous efficiency reaches 93 lm/W and showed a typical efficiency droop as the inject current increases (Fig. S23). The stability of the LED has been check after 100 working cycles and its performance remain unchanged.

## Conclusions

In conclusion, we proposed a CVD method for post-encapsulation of graphene shell layer on Cu NWs network TEs which has achieved their ohmic contact on GaN based LED chips. Ultra-long (>40 μm) and super-fine (~13 nm) Cu NWs were synthesized via a solution method. The gas phase encapsulation of graphene on Cu NWs network effectively improves the optoelectronic performances such as conductivity and antioxidant ability. Combined with photolithography and imprint techniques, Cu@graphene NW TEs could be fabricated on GaN-based LED chips on wafer. By using pre-annealing treatment, ohmic contact was achieved to both n-type and p-type GaN conducting layers before graphene encapsulation. After graphene encapsulation, a completely transparent LED without light-blocking electrodes could be obtained and successfully lit with bright blue emission. APSYS simulation and EL micrograph imaging revealed a novel scattered-point contact mode in NWs network electrode, which could produce low resistance and high injection current through the point discharge effect, especially for those Schottky contact metal-semiconductor interfaces. The Cu@graphene NWs transparent electrodes are deserved to find broad applications in the field of future optoelectronic devices.

## Experimental Section

### Chemicals

Copper(II) chloride dihydrate (CuCl_2_•2H_2_O, AR, SCRC), nickel(II) acetylacetonate [Ni(acac)_2_, 95%, Strem Chemicals Inc.], hexane (AR, SCRC) oleylamine (80~90%, Acros Organics), hydrogen (99.999%, Linde), argon (99.999%, Linde) and methane (99.999%, Kongfen) were all used as received.

### Synthesis of Cu NWs

A solution method was used to synthesize the Cu NWs, as shown in the following steps: In a typical procedure, 0.8 mmol CuCl_2_•2H_2_O and 0.4 mmol Ni(acac)_2_ were mixed with 10 ml oleylamine in a 50 ml three-necked flask and kept under a flow of high-purity argon at 80 °C for 20 min with strong magnetic stirring. After fully dissolution, the resulting solution was heated up to 175 °C and kept for 3 h. After cooling down to room temperature naturally, excess hexane was added into the red solution to give a red precipitate which was isolated via centrifugation (10000 rpm for 10 min). Cu NWs ink was obtained by washing the precipitate with a mixture of hexane and acetone, and then dispersing them into hexane by bath sonication for 10 min. Then, the Cu NWs ink was filtered onto a nitrocellulose membrane uniformly by vacuum suction to form Cu NWs percolation network. The nitrocellulose membrane which contacts intimately with Cu NWs was put onto target substrates and a boat-like stamp was used to imprint under uniform pressure for about 30 s. Finally, the nitrocellulose membrane was peeled off to leave Cu NWs network on the substrate. Such process could lead to uniform dispersion of Cu NWs network on target substrate such as Si.

### Fabrication of TEs with Cu NWs network

Thin films were percolated by vacuum filtration and transferred to target substrates such as silicon, sapphire and quartz glass. Firstly, as-synthesized Cu NWs ink was filtered onto a nitrocellulose membrane uniformly by vacuum suction to form Cu NWs percolation network. Secondly, the nitrocellulose membrane which contacts intimately with Cu NWs was put onto target substrates and a boat-like stamp was used to imprint under uniform pressure for about 30 s. Finally, the nitrocellulose membrane was peeled off to leave Cu NWs network on the substrate and thus, the TE formed on the substrate surface firmly.

### Graphene encapsulation on Cu NWs network

The graphene encapsulation was carried out by using hydrocarbons precursors in a low-pressure CVD system at around 700 °C. A mixture of CH_4_ (10~40 sccm) and H_2_ (2~10 sccm) was used for the gas precursors. The imprinted Cu NWs network on a target substrate [e.g. Si (100) or GaN (0001)] was placed in a Cu foil pocket and transferred into the quartz tube by a magnetic manipulator. This Cu NWs network was first thermally annealed under vacuum (10^−4^ Torr) at 200 °C for 20 min to remove the surfactants and achieve the nano-welding of the interlacing NWs junctions. Then, the sample was dragged away from the heating zone and the furnace was heated up to 700 °C. When reaching the growth temperature, the sample was again pushed into the heating zone for 1~5 min and meanwhile, the hydrocarbons precursors (CH_4_ and H_2_) were injected into the quartz tube for accomplishing the graphene encapsulation. After coating, the sample was immediately pulled out to the room temperature area for rapid cooling. This encapsulating and annealing process could be repeated for several circles to control the coating thickness of graphene shell layers.

### Fabrication of LED chips with Cu@graphene NW TEs

The GaN-based LED epitaxy-layers were grown on a c-plane sapphire substrate by metal-organic chemical vapor deposition (MOCVD). A 2.9 µm thick undoped GaN layer and 4 µm thick n-GaN:Si layer were grown at 1020 °C on sapphire substrate before the growth of three InGaN(3 nm)/GaN(10 nm) multiple quantum wells (MQWs) at 770 °C, and this was followed by the growth of 3 nm p-AlGaN. Then, a 200 nm thick p-GaN:Mg layer were grown at 950 °C (Fig. S24). After that, the epitaxy layers were etched by an inductively coupled plasma (ICP) etching process using Cl_2_/CH_4_/H_2_/Ar source gases in the presence of protective photoresist as an etch mask until the n-GaN layer was exposed. Then the photoresist was removed and another patterned mask was made for protecting the edges between p-GaN stage and n-GaN area (see Supporting Information, Fig. S25). To fabricate the Cu NWs networks as transparent electrodes, the Cu NWs networks was imprinted onto the prepared LED wafer and then, the mask photoresist was removed by diping in acetone for 30 min.

The as-prepared LED chips on board then was processed for achieving ohmic contact of Cu NW network and accomplishing graphene encapsulaton. The wafer was put in a Cu foil pocket and thermally annealed under vacuum (10^−4^ Torr) at 200 °C for 20 min to achieve the nano-welding and ohmic contact of the interlacing NWs junctions. Then, the sample was dragged away from the heating zone and the furnace was heated up to 700 °C. A mixture of CH_4_ and H_2_ was used for the gas precursors and the encapsulation of graphene on Cu NWs networks was then processed by pushing the sample again into the heating zone for 1~5 min. After coating, the sample was immediately pulled out to the room temperature zone for rapid cooling.

The fabrication processes are as follows: (i) after cleaning of the surface, the photoresist was spin-coated onto the GaN wafer; (ii) after short heating, the wafer aligned with a patterned mask plate was exposed to UV light to effect selective exposure on patterned area; (iii) the photoresist was developed and then cured at 100 °C to form pattern on the GaN surface; (iv) the Cu NWs network layer was imprinted onto the GaN surface by filter imprinting method; (v) the photoresist pattern was removed by heating (~50 °C) in acetone and the patterned Cu NW TEs left on the surface; (vi) nano-welding treatment was carried out at 200 °C in vacuum to achieving conducting of the Cu NWs network as well as ohmic contact to the GaN layer; and (vii) gas phase encapsulation of 3D gaphene shell layer was performed to obtain Cu@grahene NWs network.

### Characterization

The as-obtained Cu NWs, Cu@graphene NWs networks, TEs, and LED chips were characterized by means of Normarski optical microscopy (Olympus BX51M), SEM (Hitachi S-4800 scanning electron microscope operated at 20 kV), XPS (PHI Quantera XPS, using monochromatic Al Kα X-rays), Raman spectroscopy (Renishaw InVia Raman Microprobe equipped with a 532-nm laser) and TEM (TECNAI F-30 transmission electron microscope operated at 300 kV). The EDS in SEM and TEM systems was used to perform elemental mapping over the selected areas of Cu@graphene NWs. The transmittance was tested using a Varian Cary 5000 UV-Vis-NIR spectrophotometer. The sheet resistance was measured using the four-wire resistivity measurement with a Keithley 2450 system. The current-voltage (I-V) characteristics measurement was carried out on a probe station. The electroluminescence spectra were recorded via a Fiber optic spectrometer.

## Electronic supplementary material

SUPPLEMENTARY INFORMATION
