# Supplementary material for: Highly transparent light emitting diodes on graphene encapsulated Cu nanowires network
Source: Sci Rep. 2018 Sep 13;8:13721. doi: 10.1038/s41598-018-31903-7 (PMC6137134; doi:10.1038/s41598-018-31903-7)
Supplement: Supplementary file 1 — SUPPLEMENTARY INFORMATION [file 41598_2018_31903_MOESM1_ESM.pdf]

Supporting Information for :

Highly transparent light emitting diodes on graphene encapsulated Cu nanowires network

Youyang Huang[1], Zongxing Huang [1], Zhibai Zhong[1], Xu Yang[1], Qiming Hong[2], Huachun Wang [1], Shengrong Huang[3], Na Gao[1], Xiaohong Chen[1,*], Duanjun Cai[1,3,*], and Junyong Kang[1]

1Fujian Key Laboratory of Semiconductor Materials and Applications,CI center for OSED, College of Physical Science and Technology, Xiamen University, Xiamen 361005, China.

2 College of Chemistry and Chemical Engineering, Xiamen University, Xiamen 361005,China.

3 Department of Chemistry, Duke University, Durham, NC 27708-0354, U.S.A.

* Corresponding author e-mail:dcai@xmu.edu.cn， xhchen@xmu.edu.cn

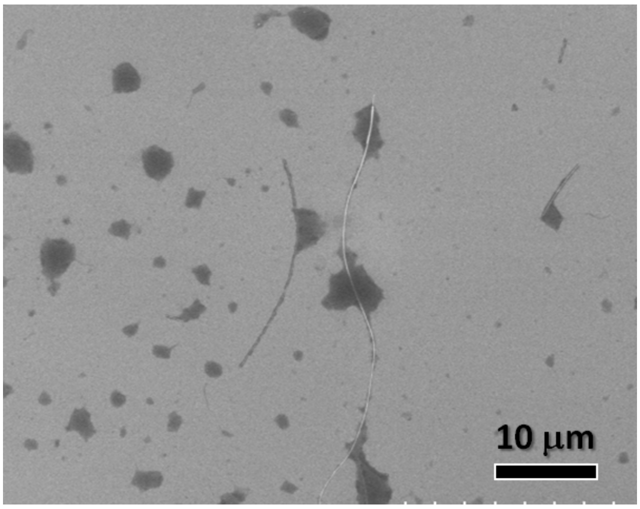

Fig. S1 SEM image of an individual Cu NW showing the length.

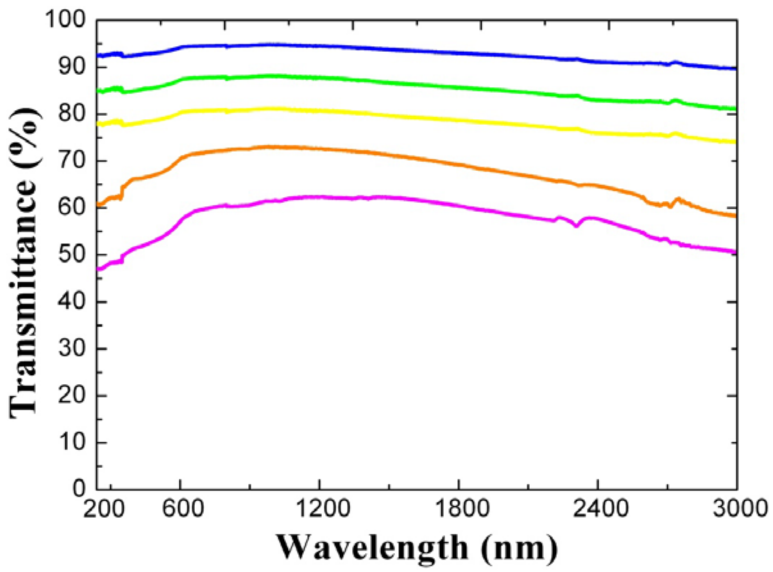

Fig. S2. UV-Vis-NIR transmission spectra for the Cu NS TEs fabricated with Cu NS inks in various concentrations. A broad transparency from 200 to 3000 nm is achieved.

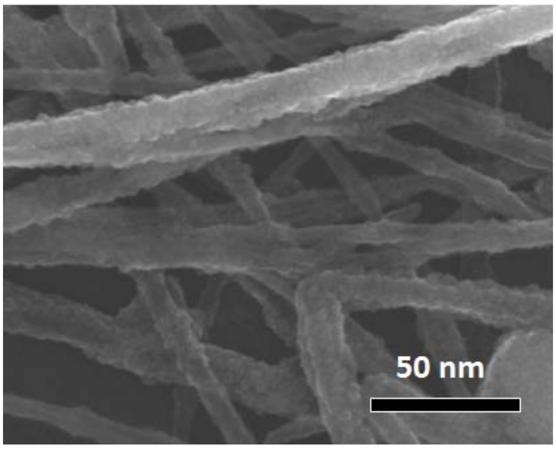

Fig. S3. SEM images of pure Cu NWs after heating at 200 °C for 10 min. The oxidation of Cu makes the NW sidewall surface seriously rough.

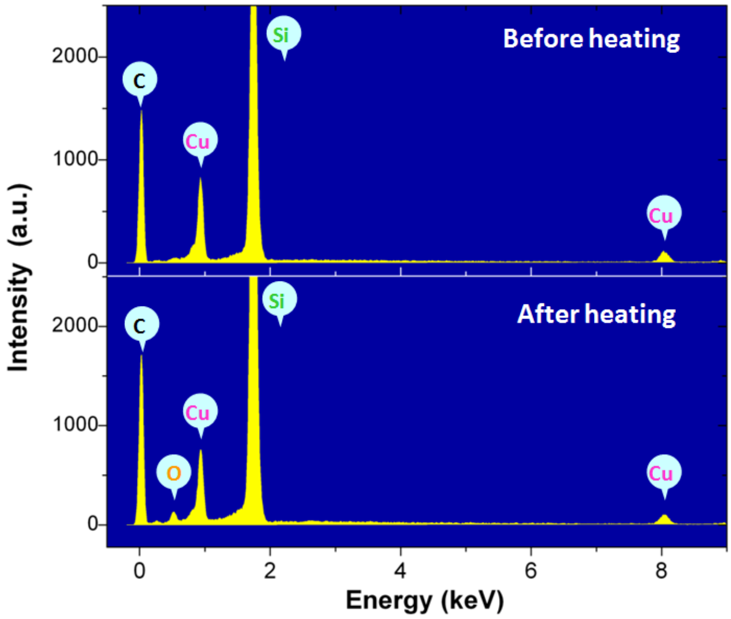

Fig. S4. EDS of Cu NWs before and after heating at 200 °C in air. Oxygen could be acquired on the surface, indicating the oxidation of the surface of Cu NWs.

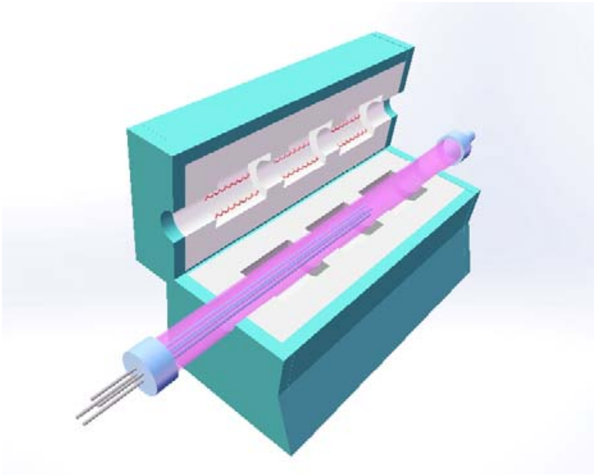

Fig.S5 Schematic of a low pressure CVD system for gas phase graphene capsulation on Cu NWs. There are three heating zones and several separated gas lines.

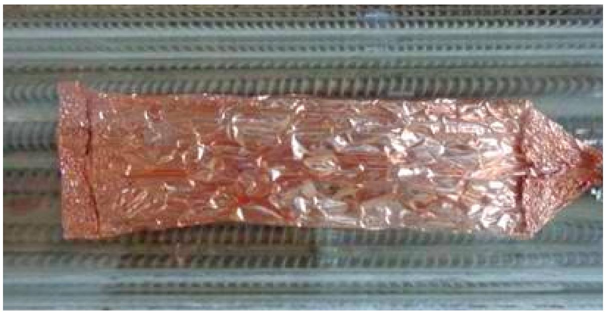

Fig. S6. Photograph of Cu pocket used for producing higher vapor pressure of Cu to protect the Cu NWs from melting under high reaction temparature.

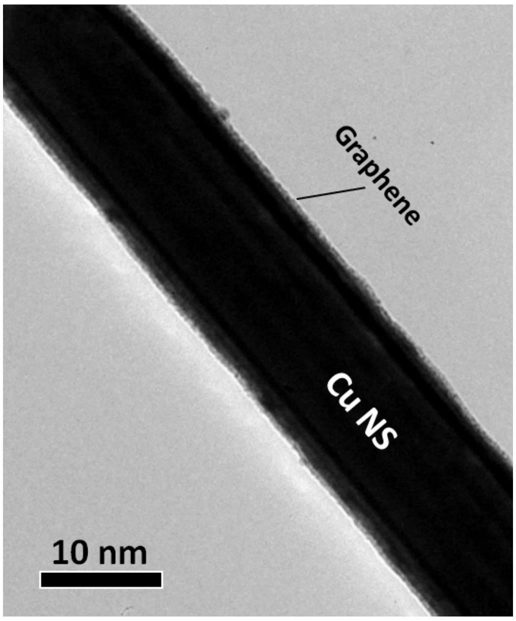

Fig. S7. Bright-field TEM image of individual graphene capsulated Cu NW. The graphene shell layer is uniformly capsulated on the sidewall surface and appears transparent.

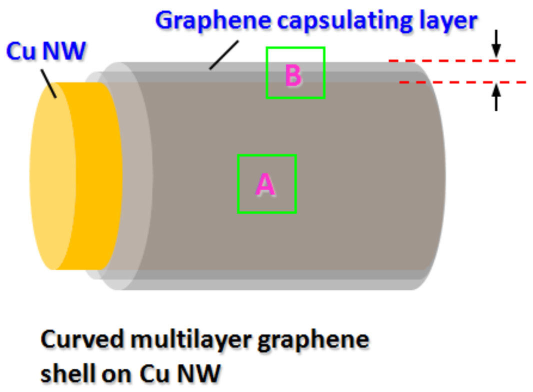

Fig. S8. Schematic of curved multilayer graphene shell capsulated on the sidewall surface of Cu NW. The A and B rectangular boxes indicate the TEM imaging from different positions.

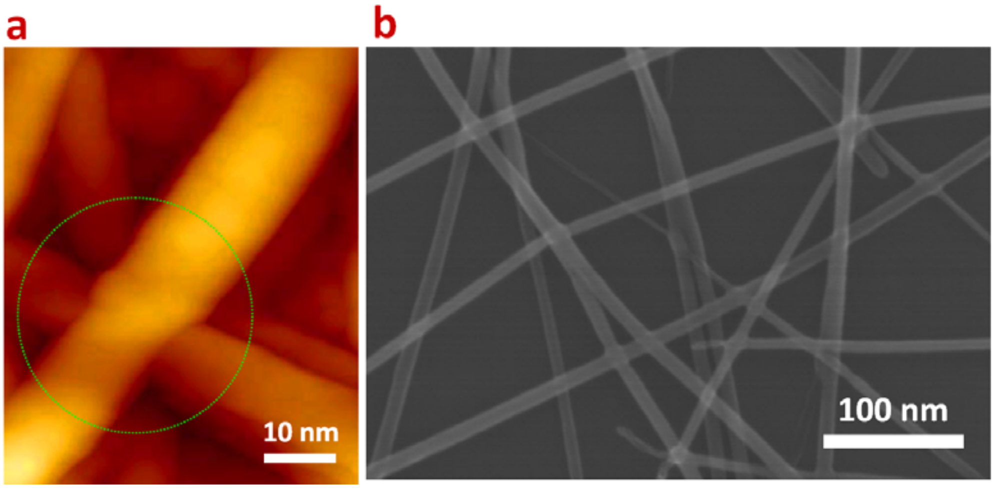

Fig. S9. (a) AFM image of junction of Cu NWs network and (b) SEM image of Cu@graphene NWs netwok with nano-welding junctions.

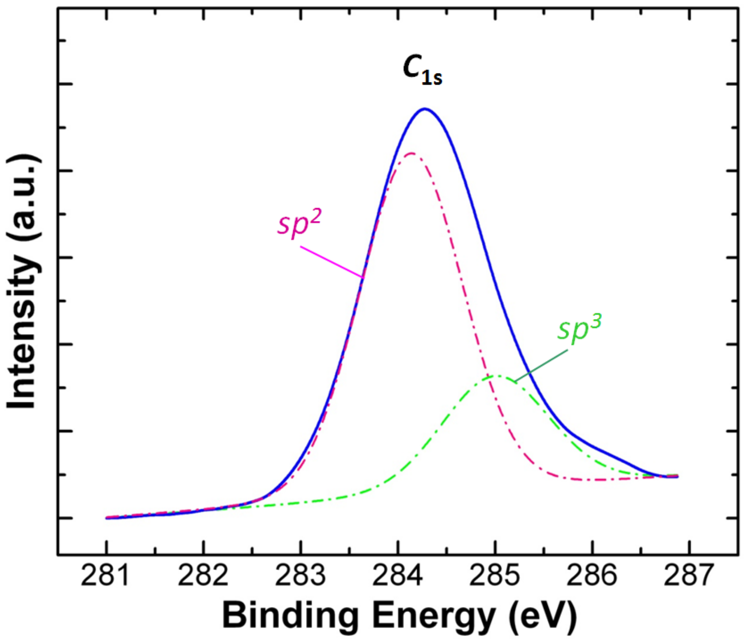

Fig. S10. XPS spectrum of Cu@graphene NWs, showing the sp^2/sp^3 hybridization ratio of C-C bonding.

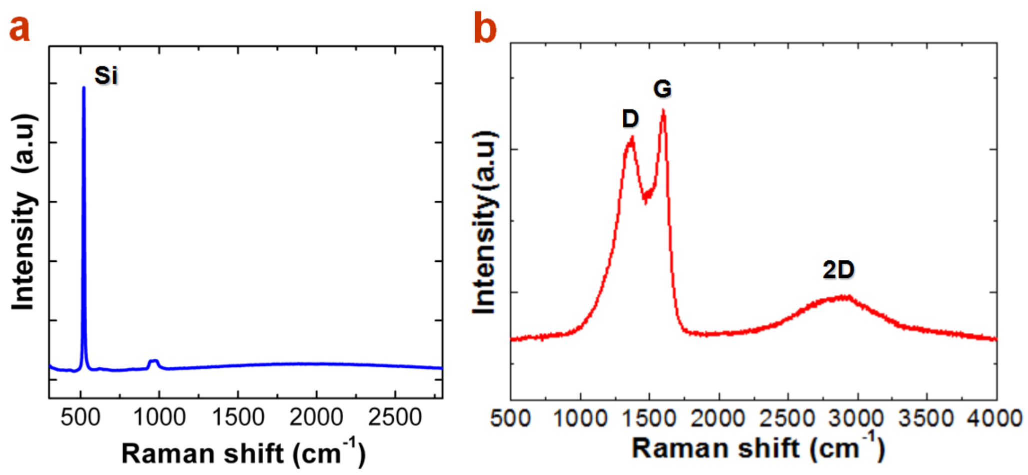

Fig. S11. Raman spectra of (a) bare Si surface and (b) Cu@graphene NWs. Clear characteristic peaks G and 2D of graphene could be distinguished. The growth of graphene is absent on the bare Si surface.

We performed the same CVD treatment for Cu NWs in vacuum without injection of methane or hydrogen (Sample#1) and conducted measurements on the morphology as well as the optoelectronic performance of Cu NWs. The control sample (Sample#2) was routinely annealed under low temperature (200 ^{o}C) for comparison. We found from SEM images (**Fig. R1**) that the average diameter of Sample#1 after short annealing (3 min) under high temperature (700 ^{o}C) becomes slightly thinner (by about 20%), agreeing well with the discussions in our manuscript. The transmittance was

improved from 70% up to 85% after high temperature annealing. About the surface smoothness, high magnification SEM images were taken for detailed analysis. The sidewall surface is extremely smooth. However, the smoothness fluctuation and improvement below 1 nm is almost undistinguishable. On the other hand, the sheet resistance of sample#1 was measured to be about 20 Ohm/sq, slightly better than the sample#2 (25 Ohm/sq). Thus, it is reasonable to believe that the further graphene capsulation on this sidewall surface is the key for the further considerable improvement of optoelectronic performance, as shown in the manuscript.

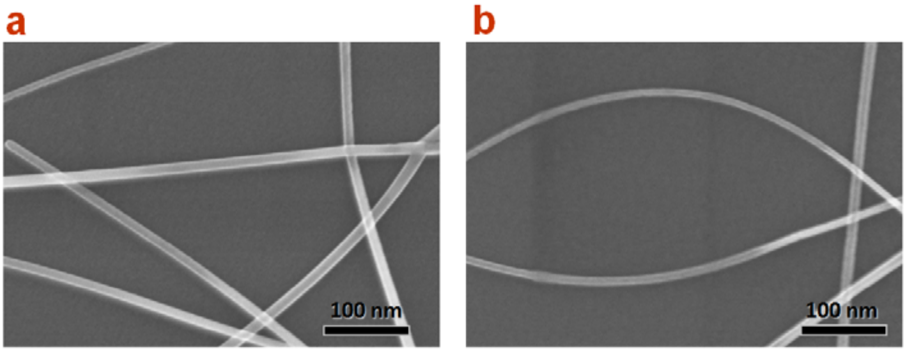

Fig. S12 SEM images of Cu NWs annealed at 200 ^{o}C (a) and 700 ^{o}C (b). The reduction of diameter at higher temperature could be observed.

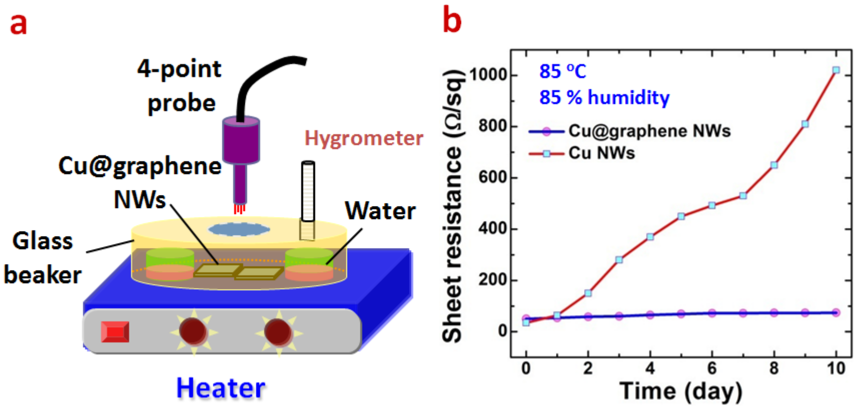

Fig. S13. (a) Schematic of the setup of stability measurements under high temperature and high humidity, and (b) sheet resistance as a function of heating time.

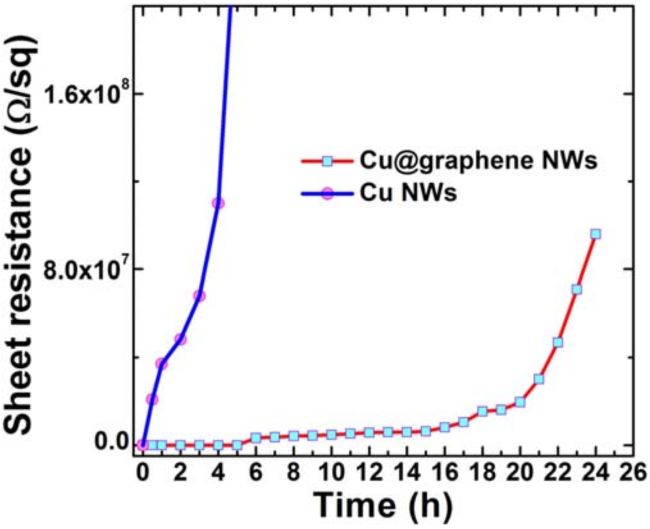

Fig. S14. Sheet resistance as a function of heating time under 200 ^oC.

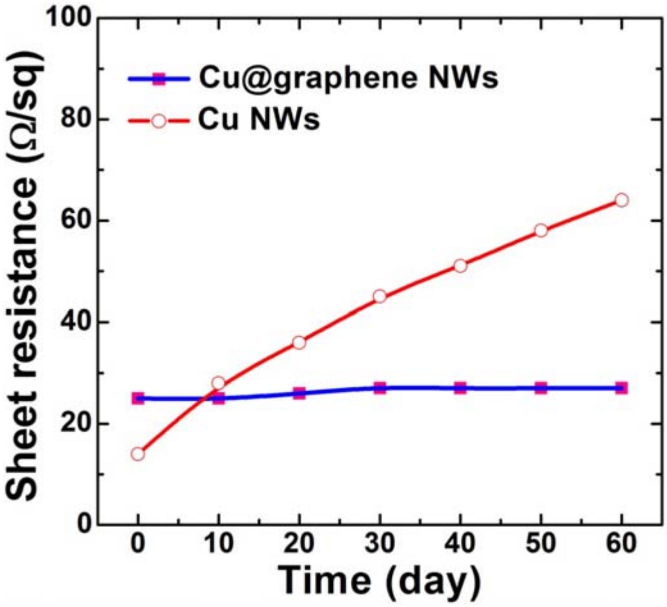

Fig. S15. Sheet resistance as a function of days for Cu NWs and Cu@graphene NWs under ambient.

(1) Fabrication of Cu@graphene NWs network on Si substrate with our standard procedure; (2) spin-coating PDMS on this Cu@graphene NWs/Si surface to make Cu@graphene NWs embedded into PDMS; (3) half curing of PDMS at room temperature or low temperature (120 ^oC); (4) peel-off of PDMS/Cu@graphene NWs film from Si surface; and (5) transfer this PDMS/Cu@graphene NWs film onto target substrates such PET or PI. By this means, the Cu@graphene NWs could be conveniently combined with those common substrates which are not suitable for high temperature treatments.

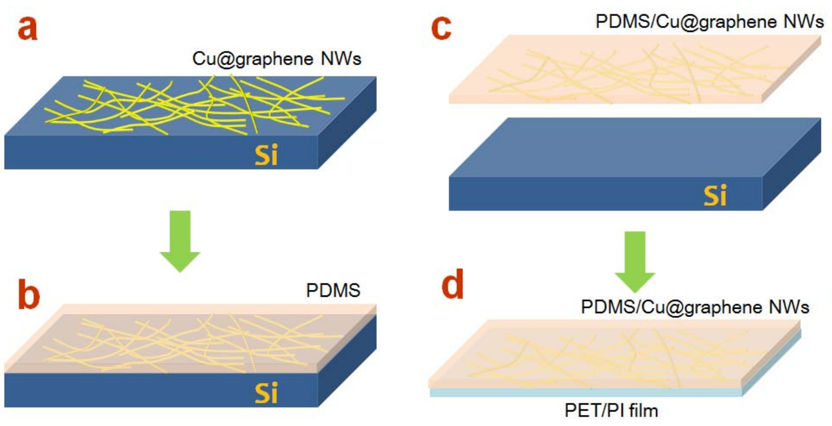

Fig. S16 Schematics of the fabrication and transfer procedure of Cu@graphene NWs by PDMS. (a) Synthesis of Cu@graphene NWs network on Si substrate, (b) coating of PDMS (or PMMA) by spin-coating method and curing at room temperature, (c) peel-off of PDMS/Cu@graphene NWs composite, and (d) transfer of PDMS/Cu@grahene NWs onto target substrate, e.g., PET or PI film.

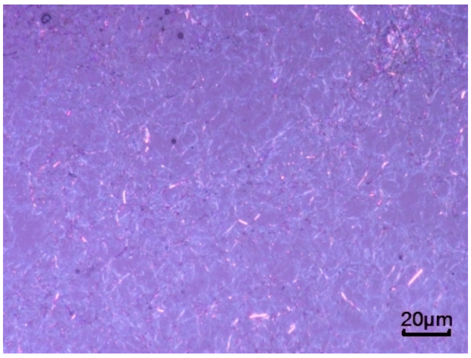

Fig. S17 Optical micrograph of PDMS/Cu@graphene NWs network peeled off from Si surface.

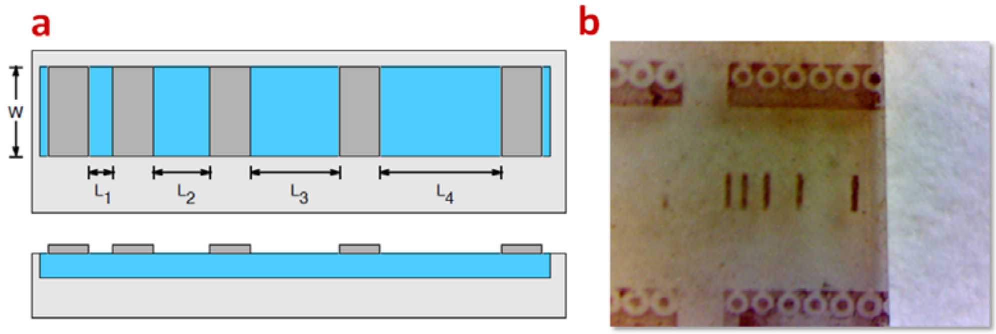

Fig. S18. TLM test pattern. (a) Schematic of pattern with electrode bar array for TLM measurements. An array of contacts (darker gray rectangular bar), with various spacings, is formed on GaN conducting layer. (b) Micrograph of TLM pattern fabricated on p-GaN epilayer surface.

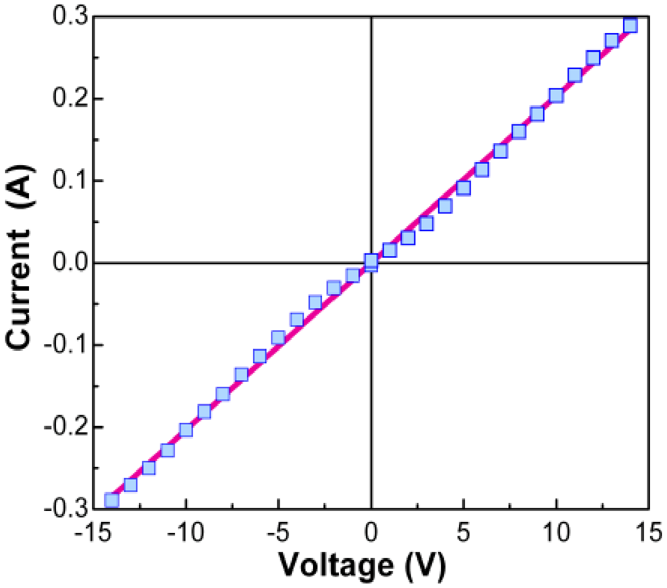

Fig. S19. I-V characteristics of the contact of Cu@graphene NW TEs to n-GaN.

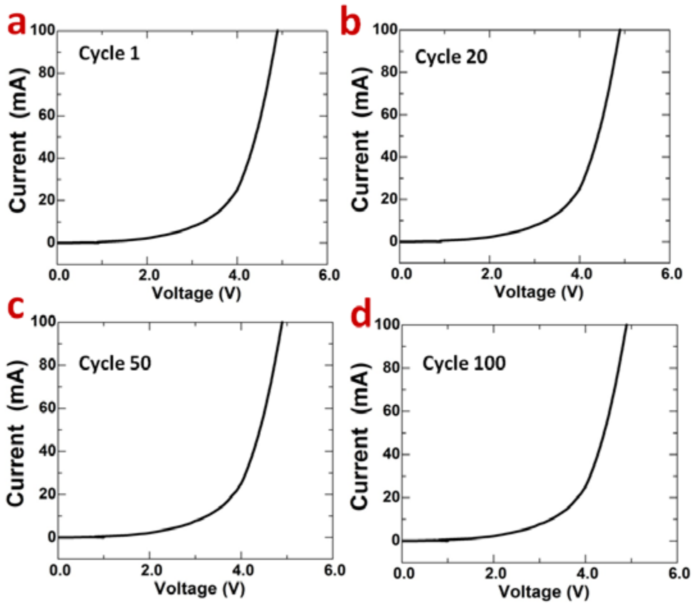

Fig. 20 I-V curves of LED measured after 1, 20, 50 and 100 cycles

In order to apply ITO on p-type GaN conducting layer on the top of LED, previously we usually added another n^+ (n-GaN) capping layer ontop p-GaN to enhance the ITO contact. However, that processing was too complicated. Now, we directly deposit ITO on p-GaN as a transparent current spreading layer and use an electrode pad (gold) for current injection. The structure and photograph of the LED chip with ITO is shown in **Fig. S21**. By this means, the LED could also work well as that with n^+ layer. The performance of this ITO LED was characterized for comparison. **Fig. S22** shows the I-V curve, which has a turn-on voltage at about 2.64 V. This is slightly higher than the Cu@graphene NW's one (2.15 V), indicating the

better contact of Cu NWs to the p-GaN layer. The optical property of ITO LED shows the similar emission wavelength (440 nm) as that of the Cu NW' one (437 nm). With TLM model, we determined the contact resistance of ITO layer to p-GaN, which is about 4.03×10^{-3} Ω cm^2.

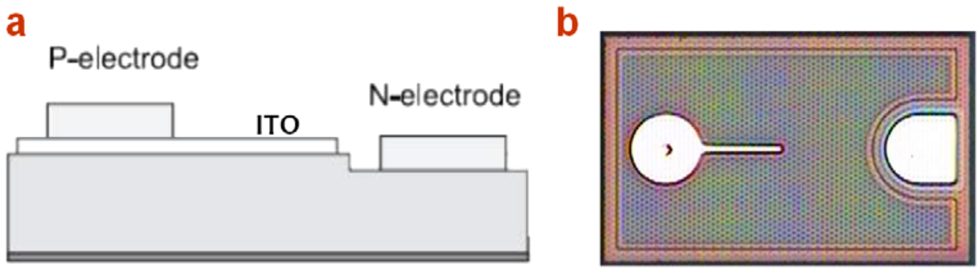

Fig. S21 LED chip with ITO as transparent current spreading layer. (a) schematic sideview of electrodes on GaN-based blue LED chip and (b) topview photograph of an individual LED chip with ITO electrode.

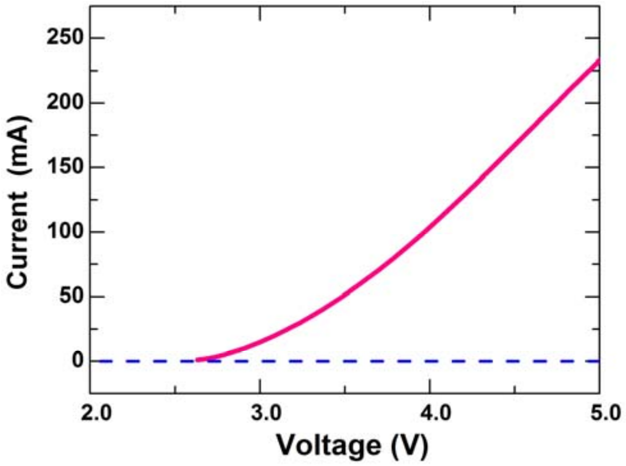

Fig. S22 I-V curve of LED with ITO transparent electrode.

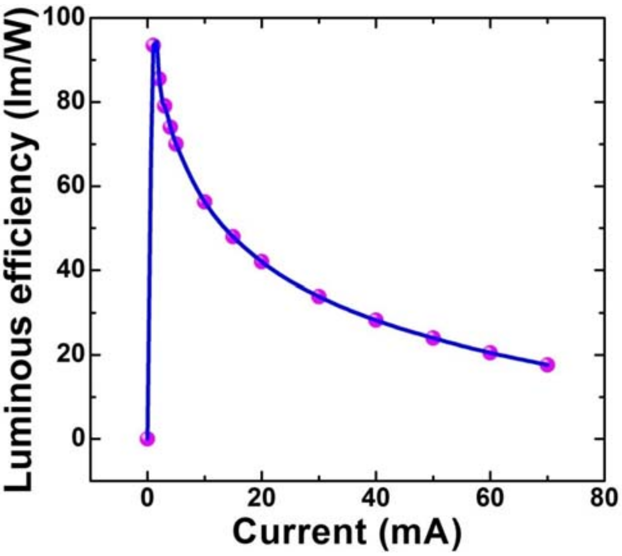

Fig. S23. Luminous efficiency as a function of current for the transparent LED.

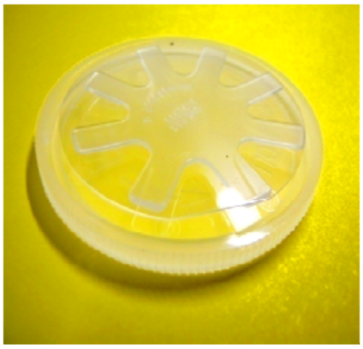

Fig. S24. GaN-based LED wafer prepared by MOCVD system (2”).

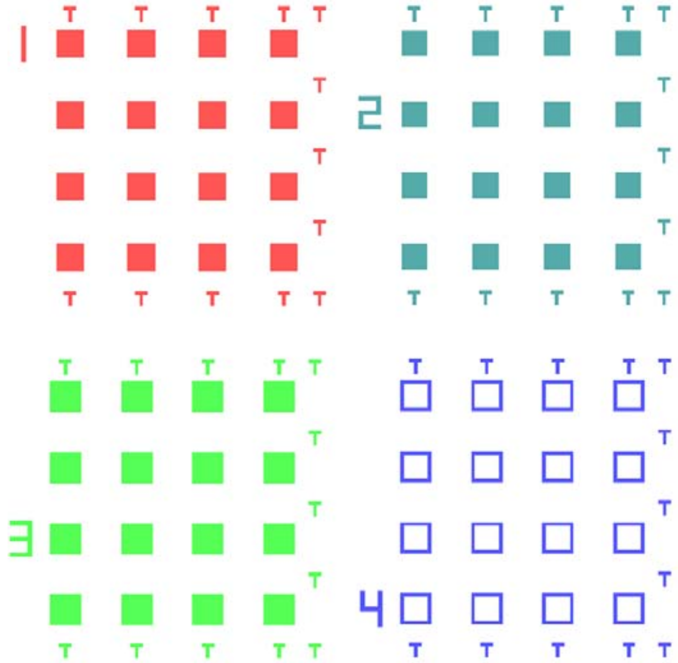

Fig. S25. A set of photoresist mask patterns for the fabrication of Cu@graphene NW TEs on LED chips. (1) Mask for etching LED chips (300 μm × 300 μm); (2) mask for fabricating uniquely p-type electrode on p-stage; (3) mask for fabricating n-type electrode on n-GaN area; and (4) mask for fabricating simultaneously n- and p-type electrodes. The edge between p-stage and n-type area is protected by the ring masks.
